# The role of PDGFRA as a therapeutic target in young colorectal cancer patients

**DOI:** 10.1186/s12967-021-03088-7

**Published:** 2021-10-26

**Authors:** Tae Won Kim, Hye Kyung Hong, Chung Lee, Sunmin Kim, Woo Yong Lee, Seong Hyeon Yun, Hee Cheol Kim, Jung Wook Huh, Yoon Ah Park, Je-Gun Joung, Woong-Yang Park, Yong Beom Cho

**Affiliations:** 1 Department of Health Sciences and Technology, SAIHST, Sungkyunkwan University, Seoul, Republic of Korea; 2 Samsung Biomedical Research Institute, Seoul, Republic of Korea; 3 Samsung Genome Institute, Samsung Medical Center, 81 Irwon-ro, Gangnam-gu, Seoul, 06351 Republic of Korea; 4 Department of Surgery, Samsung Medical Center, Sungkyunkwan University School of Medicine, 81 Irwon-ro, Gangnam-gu, Seoul, 06351 Republic of Korea; 5 Department of Biomedical Science, CHA University, Pocheon-si, South Korea; 6 Department of Molecular Cell Biology, Sungkyunkwan University School of Medicine, Seoul, Korea; 7 Department of Biopharmaceutical Convergence, Sunkyunkwan University, Seoul, Korea

**Keywords:** Young colorectal cancer, Biomarker, CMS, PDGFRA, Regorafenib

## Abstract

**Background:**

Young patients with colorectal cancer (CRC) exhibit poor prognoses compared to older patients due to the difficulty in early diagnosis and treatment. However, the underlying molecular characteristics are still unclear.

**Methods:**

We conducted a comprehensive analysis of 49 CRC patients without hereditary CRC using the whole-exome and RNA sequencing with tumor and matched normal samples. A total of 594 TCGA samples and 4 patient-derived cells were utilized for validation.

**Results:**

Consensus molecular subtype 4 (CMS4) (53.85%) and CMS2 (38.46%) were enriched in the young (≤ 40 years) and old (> 60 years) age groups, respectively. A CMS4-associated gene, platelet-derived growth factor receptor α (*PDGFRA*), was significantly upregulated in young patients with CRC (FC = 3.21, p = 0.0001) and was negatively correlated with age (p = 0.0001, R = − 0.526). Moreover, *PDGFRA* showed a positive co-expression with metastasis-related genes in young CRC patients. In vitro validation confirmed that young patient-derived cells (PDCs) showed an enriched expression of *PDGFRA* compared to old PDCs and a reduced proliferation rate by knockdown of *PDGFRA*. Furthermore, young CRC patients were more sensitive to regorafenib, a PDGFRA-targeting drug, than old CRC patients.

**Conclusions:**

Our study suggests that CRC in young patients is associated with CMS4 and PDGFRA. In addition, PDGFRA may serve potential of novel therapeutic strategies and represent a predictive biomarker of response to regorafenib for young CRC patients.

**Supplementary Information:**

The online version contains supplementary material available at 10.1186/s12967-021-03088-7.

## Background

Colorectal cancer (CRC) is one of the most common cancers and a leading cause of cancer-related deaths worldwide [1, 2]. However, studies on specific therapeutic strategies and appropriate drugs for CRC have not reported successful outcomes [3, 4]. CRC is predominantly a cancer in older adults with a median age at diagnosis of > 60 years [5, 6]. However, population-based studies have reported that CRC incidence has increased in the younger population than in those > 50 years old [5, 6]. Numerous studies have suggested that younger patients have poor prognoses with difficulty in early diagnosis compared to older patients [7–9]. In addition to the difficulty in treating young CRC patients, there are few specific treatment strategies for young CRC patients.

According to a study with a selected gene set showing several somatic mutations that tend to be frequent in CRC patients, several early-onset CRCs are caused by inherited genetic mutations [10], except for hereditary cancer syndromes [11]. A gene expression study of young patients without a family history of the disease suggested that upregulated genes, compared with healthy controls, are involved in various biological processes [12]. Based on the insufficient gene set and patient population in previous studies, the knowledge derived so far may be incomplete. Recently, high-throughput genomic and transcriptomic sequencing technologies have attracted attention as tools for detecting a large number of oncogenes [13] and quantifying gene expression levels with a high resolution [14]. Comparative analysis at the genomic and transcriptomic levels will provide greater details and insights into the molecular mechanism of young CRC.

Despite the widespread use of the technique, the CRC classification published so far revealed only similarities [15–18]. Recently, a more detailed gene expression-based classification of CRC was proposed with four biologically distinct consensus molecular subtypes (CMS) as follows [19]: CMS1 (microsatellite instability (MSI) and immune activation), CMS2 (epithelial type and marked WNT signaling), CMS3 (epithelial type and metabolic dysregulation), and CMS4 (mesenchymal type and activated TGF-β signaling). Additionally, the clinical relevance of CMS4 is associated with poor prognosis, while the others are characterized by a relatively good prognosis [19]. As the CMS allows a more detailed and accurate classification than the existing classification methods, many studies have been recently conducted to diagnose and treat CRC patients[20–23]. Therefore, the classification of individual CMS groups has great potential to advance precision medicine for CRC patients.

In this study, we conducted a comprehensive analysis of 49 CRC patients to understand the molecular characteristics of young patients through a whole-exome and RNA sequencing with tumor and matched normal samples. To identify young CRC patients’ specific biomarkers, we divided them into three groups: young, middle, and old. A comparison of these three groups could differentiate between young and old age CRC and reveal clearer molecular subtypes and genomic alterations. Our study provides an in-depth understanding of young CRC tumors and novel biomarkers.

## Methods

### Sample collection

This study was approved by the Institutional Review Board (IRB) of the Samsung Medical Center (IRB Approval No. SMC 2013-11-007-001). Written informed consent was obtained from each patient. The study subjects were 49 patients diagnosed with CRC at the Samsung Medical Center, Seoul, Korea. Tumor and matched adjacent normal tissues were obtained from surgical specimens. All patients were not treated before surgery.

### The cancer genome atlas (TCGA) colorectal cancer data sets

The gene expression profile of 594 mRNASeq colorectal adenocarcinoma (COAD) samples from cBioPortal (https://www.cbioportal.org/, TCGA, PanCancer Atlas) was utilized for validation.

### Isolation of genomic DNA and RNA

Genomic DNA and RNA in tissues were purified using the AllPrep DNA/RNA Mini Kit (Qiagen, Valencia, CA, USA). Genomic DNA from peripheral blood was extracted using the QIAamp DNA Blood Mini Kit (Qiagen). The genomic DNA concentration and purity were measured using a NanoDrop 8000 UV–Vis Spectrometer (Thermo Scientific Inc., Wilmington, DE, USA) and a Qubit 2.0 Fluorometer (Life Technologies Inc., Grand Island, NY, USA). DNA degradation was estimated by measuring median DNA size and ΔCt values with a 2200 TapeStation Instrument (Agilent Technologies, Santa Clara, CA, USA) and real-time PCR (Agilent Technologies), respectively. For RNA, the concentration and purity were measured with the NanoDrop and Bioanalyzer (Agilent Technologies).

### Whole-exome sequencing

Genomic DNA (1 µg) from each sample was sheared using the Covaris S220 (Covaris, Woburn, MA, USA), and a library was constructed using SureSelect XT Human All Exon V5 and a SureSelect XT Reagent Kit, HSQ (Agilent Technologies). The kit captures 335,756 exons of 21,058 genes, covering ~ 71 Mb of the human genome. After enriched exome libraries were multiplexed, the libraries were sequenced on a HiSeq 2500 platform (Illumina, San Diego, CA, USA). Briefly, a paired-end DNA sequencing library was prepared by gDNA shearing, end-repair, A-tailing, paired-end adaptor ligation, and amplification. After hybridization of the library with bait sequences for 16 h, the captured library was purified and amplified with an index barcode tag, and the library quality and quantity were measured. Sequencing was performed in the 100-bp paired-end mode of the TruSeq Rapid PE Cluster Kit and TruSeq Rapid SBS Kit (Illumina).

### Exome-seq data analysis

Raw sequencing reads were aligned to the UCSC hg19 reference genome (downloaded from http://genome.ucsc.edu), using the Burrows–Wheeler Aligner (BWA) [24] version 0.7.5a with default settings. PCR duplicates were marked using Picard-tools-1.93 (http://picard.sourceforge.net/), data cleanup was performed with GATK, and variants were identified with GATK-3.5 [25]. Point mutations were identified using MuTect [26] with paired samples. ANNOVAR [27] was used to annotate variants. Somatic copy number alterations were identified using the Excavator tool [28]. Regions of the genome that were significantly amplified or deleted across samples were detected by the GISTIC tool [29].

### RNA sequencing

Library construction for RNA sequencing was carried out with a Truseq RNA Sample Preparation v2 Kit (Illumina). Isolated total RNA was used in a reverse transcription reaction with poly (dT) primers, using SuperScriptTM II Reverse Transcriptase (Invitrogen/Life Technologies). Briefly, an RNA sequencing library was prepared by cDNA amplification, end-repair, 3′ end adenylation, adapter ligation, and amplification. Library quality and quantity were measured using the Bioanalyzer and Qubit. Sequencing of the RNA library was performed on the 100-bp paired-end mode of the TruSeq Rapid PE Cluster Kit and the TruSeq Rapid SBS Kit (Illumina).

### RNA-seq data analysis

Reads from the FASTQ files were mapped to the hg19 human reference genome using STAR aligner version 2.5.0a [30] and read counts for each gene were normalized as transcripts per million (TPM) using the RSEM program [31]. Differentially expressed genes (DEGs) were identified using the DESeq R package [32] with a cut-off (|log2 fold-change|> 2 and false discovery rate (FDR) < 0.001) [33]. DEGs were mapped to the pathway using the ReactomeFI Cytoscape [34]. CMS calls in CRC cohorts were predicted based on the gene expression profile using the R package CMSclassifier [19].

### Organoid culture

The organoid culture medium was refreshed every 2 days. For the organoid passage, BME was broken up by pipetting, and organoids were collected in a tube. The organoids were centrifuged at 1000 rpm for 3 min, and the medium was removed. Next, 5 mL of Triple Express (Invitrogen) was added, and the organoids were incubated at 37 °C for approximately 5 min. Every minute, a visual check was performed to verify the size of organoids. Care was taken not to treat the organoids too long with the Triple Express. FCS and medium were added, and the cells were spun down at 1500 rpm for 3 min. The pellet was taken up in BME, and the cells were plated in droplets of 5–10 μL each. After allowing the BME to solidify, HICS minus Wnt, both supplemented with 10-µM LY27632, was added to the plates, and organoids were incubated at 37 °C [35].

### Cell transfection

For transient transfection assays, PDGFRA siRNAs (#1: 5′ CCUCUAUCCUUCCAAAUGAUU 3′, 5′ UCAUUUGGAAGGAUAGAGGUU 3’, #2: 5′ CCGUCAAAGGAAAGAAGUUUU 3′, 5′ AACUUCUUUCCUUUGACGGUU 3′, Genolution, Seoul, Korea) were transfected into PDCs using Lipofectamine RNAiMAX (Invitrogen, Carlsbad, CA, USA) according to the manufacturer’s protocol.

### Cell proliferation assay

Cell proliferation was measured in triplicate using a colorimetric assay that determines cellular viability by evaluating the metabolic conversion based on the quantification of the ATP present using CellTiter-Glo (Promega, Madison, WI, USA). The viability of the colon cancer cells was assessed at various times, and assays were performed by adding CellTiter-Glo directly to the culture wells and incubating for 30 min at room temperature. Luminescence was measured using a Mithras microplate reader (Berthold-bio, Bad Wildbad, Germany). Three different experiments were performed for each experimental condition.

### Real-time quantitative polymerase chain reaction (RT-qPCR) analysis

Total RNA was extracted from the patient’s tissue (RNAprep Mini kit, Qiagen), and 500 ng RNA was subjected to reverse transcription using a reverse transcription kit (Bioneer). Real-time quantitative PCR amplification was performed with SYBR Green (ABI) in a real-time system (ABI, USA). Human-specific PCR primers (Bioneer) were used to analyze the expression of the following genes: PDGFRA and GAPDH. The mRNA levels of the specific genes were calculated as ΔΔCt and normalized to those of *GAPDH*.

### Cell lysis and Western blot analysis

To prepare the whole-cell extract, cells were lysed using a Pro-prep buffer (Intron Biotechnology, Seoul, Korea) containing protease inhibitors. A total of 10–40 μg of protein extract was resolved by SDS-PAGE and transferred to polyvinylidene fluoride (PVDF) membranes. The membranes were probed with primary antibodies against PDGFRA (ab65258, Abcam) and β-actin (#3700, Cell Signaling Technology) followed by incubation with secondary antibodies conjugated to horseradish peroxidase (Santa Cruz Biotechnology, CA, USA). β-actin was used as a loading control in the western blot analysis.

### High-throughput screening (HTS)

The chip layout was designed for drug screening in a single micropillar chip. In the micropillar chip, ~ 80–100 cells were immociliized with 0.75% alginate. We tested 67 drugs in CRC PDCs. Among them, we revealed regorafenib data in this study. A 50-nL droplet of a 1:1 cell mixture of 1.5% alginate and 950-nL droplet of 3D culture media was dispensed with the ASFA Spotter ST (MBD). After overnight incubation, a 950-nL droplet of the drugs was dispensed with the ASFA Spotter ST and stamped with the micropillar chip containing the cells. The combined chips were incubated for 5 days at 37 °C and 5% CO_2_ in an incubator for the cell viability assay. After incubation, the micropillar chips were stained with a staining buffer (MBD-STA50, Medical and Bio Device) in a Calcein-AM (Invitrogen, live-cell starting dye) for 1 h in the dark at room temperature. The stained micropillar chips were washed with a staining buffer for 30 min and dried in the dark.

### Statistical analysis

Statistical comparisons were performed using GraphPad Prism. Data are expressed as the mean ± standard deviation (SD). P-values < 0.05 were considered statistically significant (*P < 0.05, **P < 0.01, or ***P < 0.001).

## Results

### CMS classification according to age of CRC patients

**Fig. 1:**
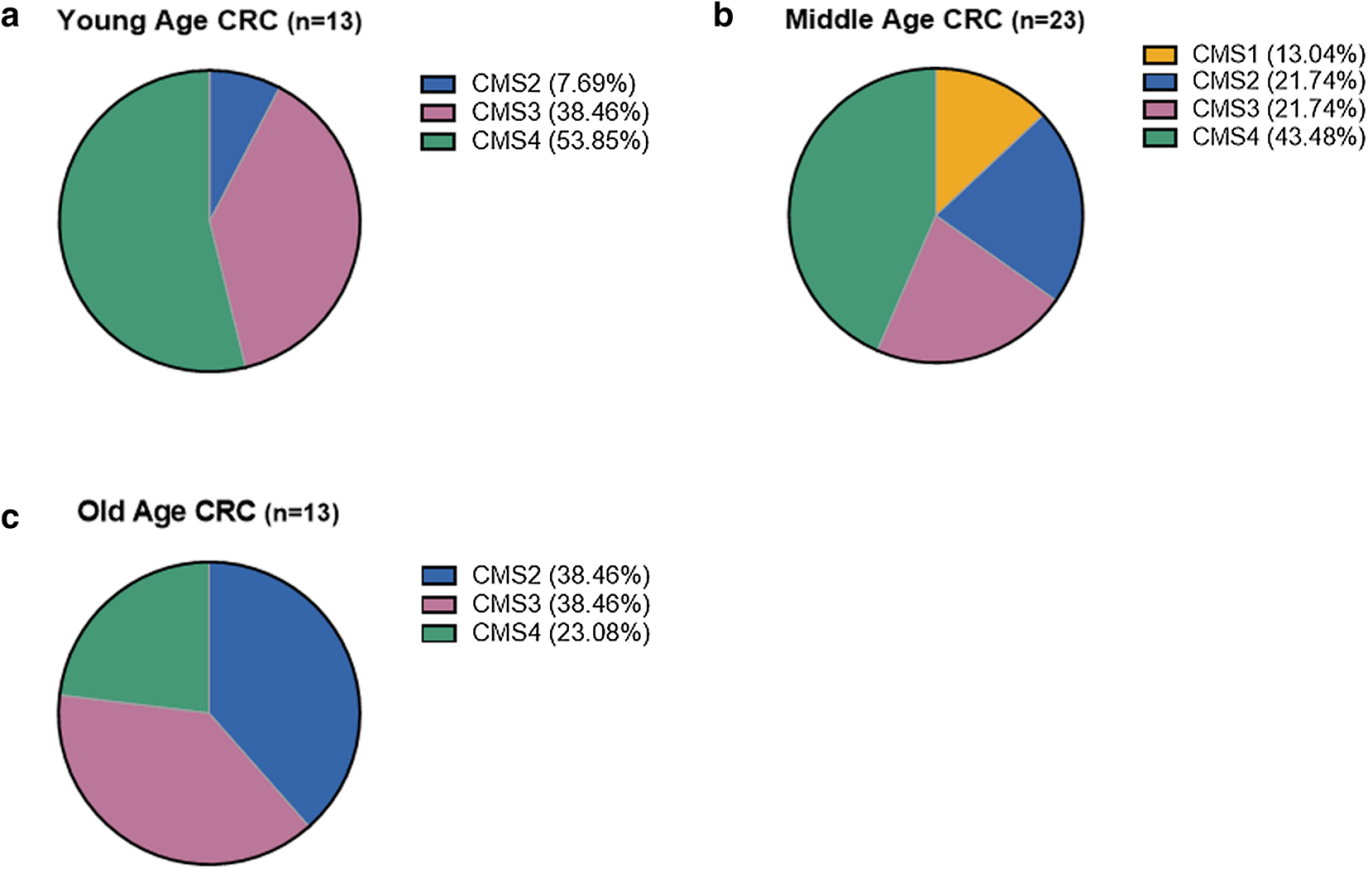
Differences in CMS type based on age. Pie chart of CMS types for three categories according to age: **a** young age group (< 40 years; *n* = 13), **b** middle-aged group (41–60 years; *n* = 23), and (c) old age group (> 60 years; *n* = 13)

To clarify the difference in the molecular characteristics between young and old patients, we divided the samples into three groups according to the age of 49 CRC patients (Table 1). The young (*n* = 13), middle-aged (*n* = 23), and old age (*n* = 13) groups included patients < 40 years old, between 41 and 60 years old, and > 60 years old, respectively. After categorizing the patient groups, CRC patients were classified according to CMS using gene expression profiles [19]. In particular, CMS4 (53.85%) was the most frequent in young CRC, followed by CMS3 (38.46%) and CMS2 (7.69%). CMS1 was absent in the young age group (Fig. 1a). In the middle CRC group, the order of the frequencies was the same as in the young age group, but the frequencies of CMS2 (21.74%) and CMS1 (13.04%) were higher (Fig. 1b). In the old CRC group, the frequency of CMS4 (23.08%) was the lowest, while CMS2 (38.46%) and CMS3 (38.46%) showed the same frequency (Fig. 1c). Thus, CMS4 frequency gradually decreased with age, while CMS2 was more frequent in the old age group (Fig. 1 a-c). Taken together, these results indicate that young CRC patients are closely associated with CMS4, which has been considered as the worst prognosis type [19]. On the other hand, young CRC patients are less related to CMS2, which has a better prognosis than the other CMSs [19].

**Table 1:** Demographic and clinical landscape of 49 CRC patients

	Young CRC	Middle CRC	Old CRC
Number	13	23	13
Age	< = 40	41 – 60	> 60
Sex			
Male	5 (38.5%)	8 (34.8%)	10 (76.9%)
Female	8 (61.5%)	15 (65.2%)	3 (23.1%)
Tumor location			
Colon	9 (69.2%)	19 (82.6%)	12 (92.3%)
Rectum	4 (30.8%)	4 (17.4%)	1 (7.7%)
Cell differentiation			
Adenocarcinoma			
W/D	2 (15.4%)	1 (4.3%)	3 (23.1%)
M/D	10 (76.9%)	18 (78.4%)	9 (69.2%)
P/D	0 (0%)	3 (13.0%)	0 (0%)
Mucinous carcinoma	1 (7.7%)	1 (4.3%)	1 (7.7%)
Stage			
II	2 (15.4%)	6 (26.1%)	1 (7.7%)
III	4 (30.8%)	2 (8.7%)	3 (23.1%)
IV	7 (53.8%)	15 (65.2%)	9 (69.2%)
MSI status			
MSS	13 (100%)	20 (87%)	13 (100%)
MSI	0 (0%)	3 (13%)	0 (0%)

### Differential mRNA expression and gene profiling between young and old CRC

**Fig. 2:**
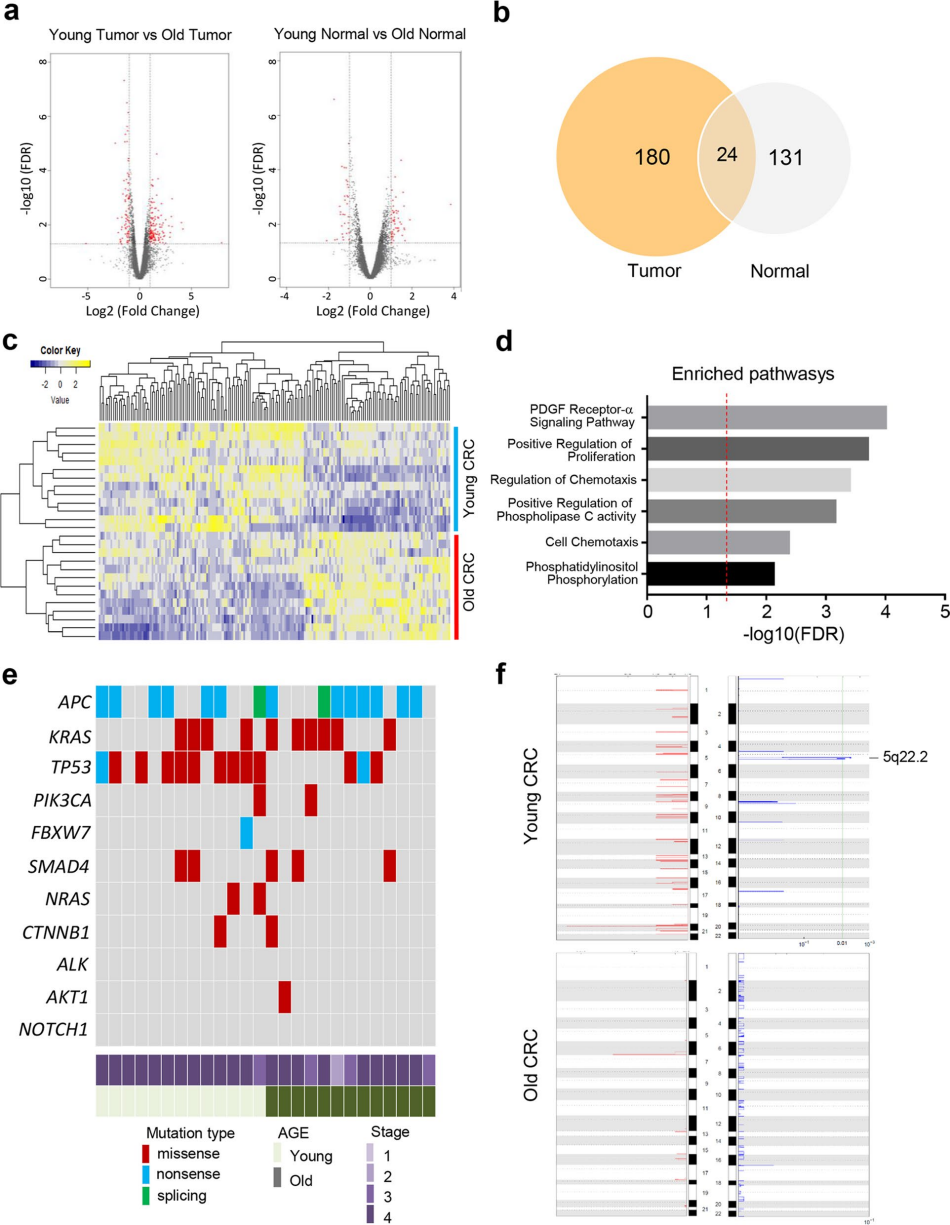
Differential mRNAs expression and gene profiling between young CRC and old CRC tissue. **a** Volcano plot of differentially expressed genes. Significant genes are highlighted in red. Dotted lines indicate cutoff values of log2 fold change (FC) ≥  ± 1, FDR ≤ 0.05. **b** Venn diagram of differentially expressed genes between young and old age groups. The number of genes obtained after a comparison with normal samples (right) and with tumor samples (left). **c** Heatmap of the expression profile of selected genes. The expression values were normalized by a z-score transformation across samples. **d** Enriched pathways from significantly changed genes in expression. **e** Oncoprint of CRC-associated mutations (missense, nonsense, and splicing). **f** Copy number amplification and deletions that are frequently detected in the samples. The green solid line indicates the q-value cutoff = 0.01

To systematically identify specific gene expression patterns related to young CRC, we detected differentially expressed genes (DEGs) from RNA sequencing data of 49 CRC tissues and their paired adjacent normal tissues. We first identified age-related genes showing a significant difference between normal samples of the young and old age groups (Fig. 2a right panel, log2 fold change (FC) ≥  ± 1, FDR ≤ 0.05). In addition, DEGs in the tumor tissues were obtained by comparing both groups using tumor samples (Fig. 2a left panel, log2 FC ≥  ± 1, FDR ≤ 0.05). Therefore, 155 genes from the DEGs in normal samples and 204 genes in the tumor were obtained. Finally, by eliminating 155 age-related genes in the normal samples, we considered a total of 180 genes as age-related DEGs specific to cancer (Fig. 2b). Among them, 105 genes were significantly upregulated and the other 75 were downregulated (Fig. 2c, log2 fold change ≥  ± 1, *p*-value ≤ 0.05, FDR ≤ 0.05). To identify cancer-related genes to be considered biomarkers of young CRC patients, we extracted only oncogenes [36] and tumor suppressor genes[37]. A number of oncogenes and tumor suppressor genes were highly upregulated or downregulated in young CRC patients (eight and four upregulated genes, and six and five downregulated genes, respectively) (Tables 2 and 3). Next, we identified the significantly changed pathways (34 upregulated and 26 downregulated) from DEGs using the network-based pathway analysis tool [38] (Additional file 1: Fig. S1). Many CMS4-related pathways [19] were enriched in young CRC patients, including PDGFRA signaling, positive regulation of cell proliferation by VEGF-activated platelet-derived growth factor (PDGF) receptor signaling, cell chemotaxis, regulation of chemotaxis, positive regulation of phospholipase C (PLC) activity, and phosphatidylinositol phosphorylation (Fig. 2d). Notably, these pathways are closely related to metastasis, angiogenesis, proliferation, and chemoresistance, which are related to CMS4 [19] and are associated with PDGFRA.

**Table 2:** Upregulated oncogenes and tumor suppressor genes in young CRC

Gene	Fold change	*q*-value	Cancer associated	Full Name
*WNT11*	4.33	0.012	Suppressor gene	Wnt family member 11
*HSPB7*	3.28	0.023	Suppressor gene	Heat shock protein family B (small) member 7
*FBLN1*	3.14	0.001	Suppressor gene	Fibulin 1
*CNN1*	2.94	0.037	Suppressor gene	Calponin 1
*CENPW*	2.34	0.021	Oncogene	Centromere protein W
*LCK*	2.3	0.032	Oncogene	LCK proto-oncogene, Src family tyrosine kinase
*CLU*	2.24	0.01	Suppressor gene	Clusterin
*AQP1*	2.23	0.033	Oncogene	Aquaporin 1
*S100A4*	2.22	0.019	Oncogene	S100 calcium binding protein A4
*TAGLN*	2.22	0.031	Suppressor gene	Transgelin
*GREM1*	2.18	0.01	Oncogene	Gremlin 1, DAN family BMP antagonist
*PDGFRA*	2.18	0.001	Oncogene	Platelet derived growth factor receptor alpha
*MMP12*	2.18	0.028	Oncogene	Matrix metallopeptidase 12
*SMO*	2	0.028	Oncogene	Smoothened, frizzled class receptor

**Table 3:** Downregulated oncogenes and tumor suppressor genes in young CRC

Gene	Fold change	*q*-value	Cancer associated	Full name
*ZIC2*	0.23	0.006	Oncogene	Zic family member 2
*PPP1R1B*	0.4	0.009	Suppressor gene	Protein phosphatase 1 regulatory inhibitor subunit 1B
*NDRG2*	0.43	0.001	Suppressor gene	NDRG family member 2 [Homo sapiens
*FEZF1*	0.44	0.039	Oncogene	FEZ family zinc finger 1 [Homo sapiens
*NFIB*	0.48	0.001	Oncogene	Nuclear factor I B
*ZNF292*	0.48	0.001	Suppressor gene	Zinc finger protein 292
*PHLDA2*	0.48	0.001	Suppressor gene	Pleckstrin homology like domain family A member 2
*CEACAM6*	0.49	0.021	Oncogene	Carcinoembryonic antigen related cell adhesion molecule 6
*CEACAM1*	0.49	0.001	Suppressor gene	Carcinoembryonic antigen related cell adhesion molecule 1

We also identified numerous somatic mutations, including missense, nonsense, and splicing mutations in primary colon tumors of CRC patients (Fig. 2e). The mutation profile showed that a *TP53* mutation was more frequent (p = 0.0029) in young CRC (76.9%, 10/13) than in old CRC (23%, 3/13) (Fig. 2e). A comparison of the copy number amplified and deleted genomic regions between both groups (Fig. 2f) showed that the copy number at chromosome 5q22.2 was significantly deleted only in young CRC samples (*q*-value < 0.01).

### High level of PDGFRA expression in young CRC patients

**Fig. 3:**
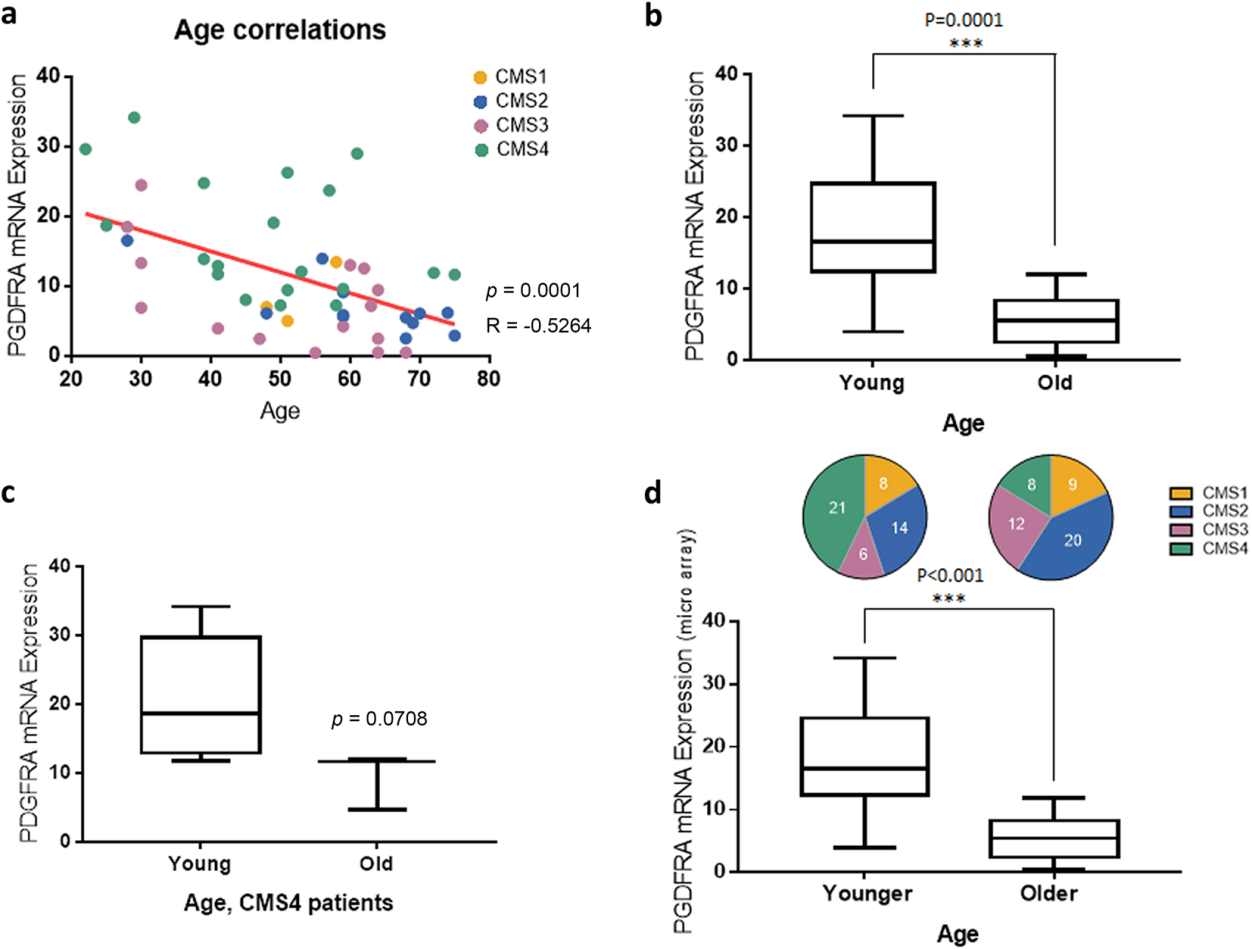
*PDGFRA* expression in young and old CRC patients. **a** Scatter plot of *PDGFRA* mRNA expression and age. Each point indicates a sample of the patient, distinguished according to CMS types. **b** Boxplot between *PDGFRA* expression levels in the young CRC group and old CRC group. **c** Comparison of *PDGFRA* expression levels in CMS4 patients. **d** Pie chart of CMS types for three categories according to age group (upper panel), and boxplot between PDGFRA expression levels in the young CRC group and old CRC group (lower panel)

To investigate whether PDGFRA is correlated with the age of CRC patients, its correlation pattern with age was examined. PDGFRA expression was negatively correlated with the age of CRC patients in our cohort (Fig. 3a, Spearman’s correlation coefficient; *P* = 0.0001, *R* = − 0.5264). Additionally, TCGA data consistently showed an inverse correlation between its expression and the age of CRC patients (Additional file 2: Fig. S2a, *P* = 0.0113, *R* = − 0.1697). Figure 3b shows that the expression of PDGFRA in the young CRC group was significantly higher than that in the old CRC patient group (*P* = 0.0001). The TCGA data revealed a similar pattern (Additional file 2: Fig. S2b, *P* = 0.2796), although this data had a limitation due to the small number of young CRC patients. To confirm whether PDGFRA was a young CRC factor rather than a CMS4 factor, its expression was compared between both groups within samples of the CMS4 type. The expression of PDGFRA in the young CRC group was higher than that in the old CRC group, even when considering only the CMS4 type (Fig. 3c, P = 0.0708). There was no significant difference due to the small sample size. To confirm our results, we also used a publicly available gene expression data set (GSE14333) derived from the primary CRC tissue (n = 188). To set similar conditions as our data, we divided it into two groups according to percentile (young CRC group: less than approximately 25th percentile, < 59 years old (n = 49) and old CRC group: greater than approximately 75th percentile, > 76 years old (n = 49)). With the CMS classification according to age, CMS4 significantly increased in the younger group (Fig. 3d, upper panel). The expression of PDGFRA in the younger group was significantly higher than that in the older group (*P* < 0.001) (Fig. 3d, lower panel).

### Co-expression of PDGFRA with a CMS4 related factor

**Fig. 4:**
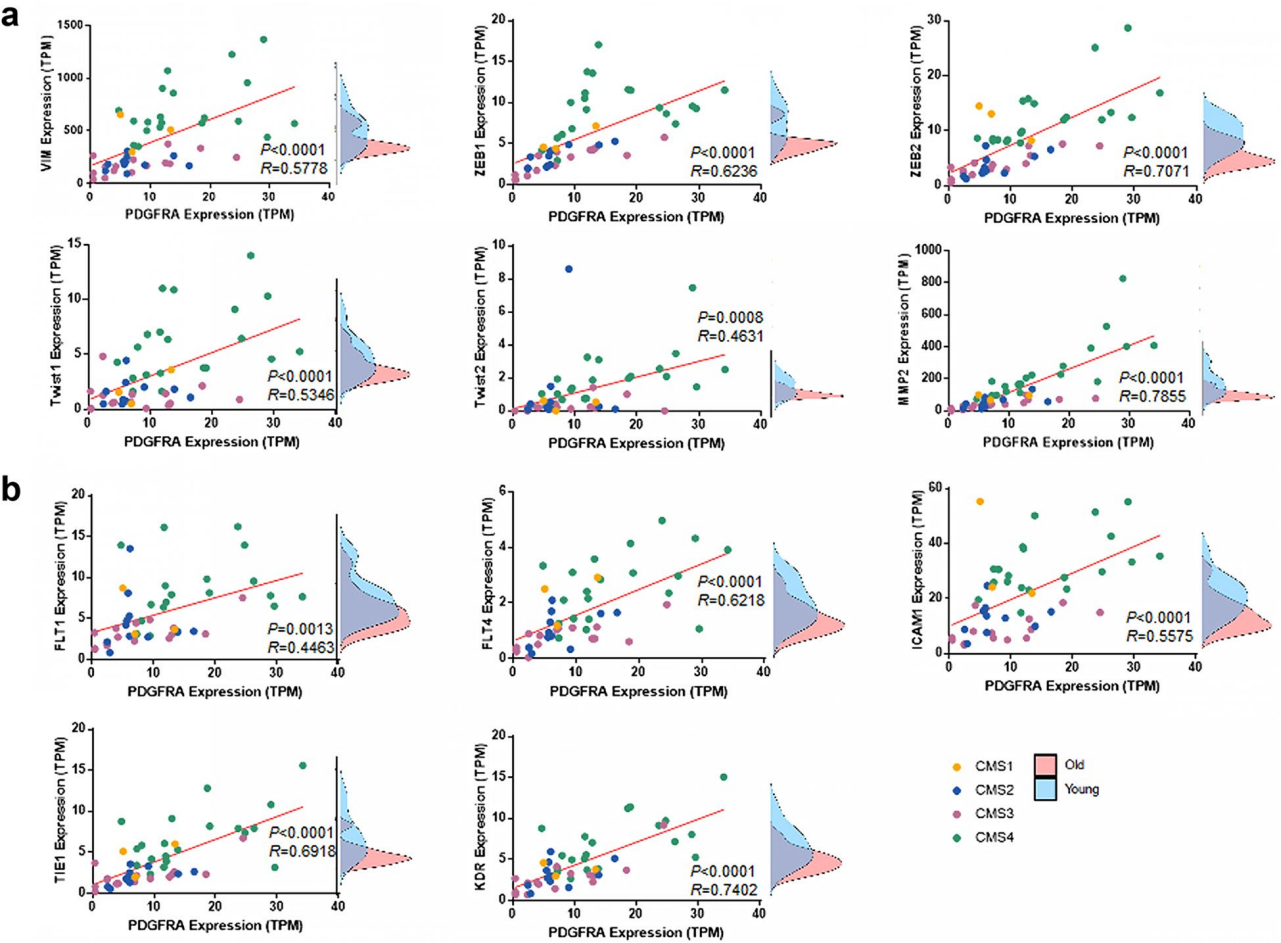
Co-expression of EMT and angiogenesis factor. **a** Scatter plot of gene expression levels between PDGFRA and EMT-associated signatures. **b** Scatter plot of gene expression levels between PDGFRA and angiogenesis-associated signatures. Each point represents a sample that is colored according to the CMS type. The comparison of expression levels for the young and old groups is shown by a density plot

CMS4 is a mesenchymal subtype of CRC and is activated in stromal invasion and angiogenesis [19]. We examined whether PDGFRA was closely associated with epithelial-mesenchymal transition (EMT) markers [39] and angiogenesis markers [40]. Among the EMT markers, six markers, namely *Vim*, *ZEB1*, *ZEB2*, *Twist1*, *Twist2*, and *MMP2*, showed positive co-expression with PDGFRA in our data set (Fig. 4a). Their expression levels were also different than the two groups, showing separate distributions. As with PDGFRA, the samples identified as CMS4 had a high expression. Among the angiogenesis markers, five markers, including *FLT1*, *FLT4*, *ICAM1*, *TIE1*, and *KDR*, showed a positive correlation in their gene expression (Fig. 4b). These markers also showed the same pattern as EMT markers. In the TCGA data, these EMT and angiogenesis markers also showed the same results as our sequencing data (Additional file 3: Fig. S3a, b). Each marker positively correlated with PDGFRA and showed a negative correlation with age (Additional file 3: Fig. S3c, d) and a co-expression pattern among each other (Additional file 3: Fig. S3e).

### PDGFRA expression and PDGFRA-targeted drug response in PDC

**Fig. 5:**
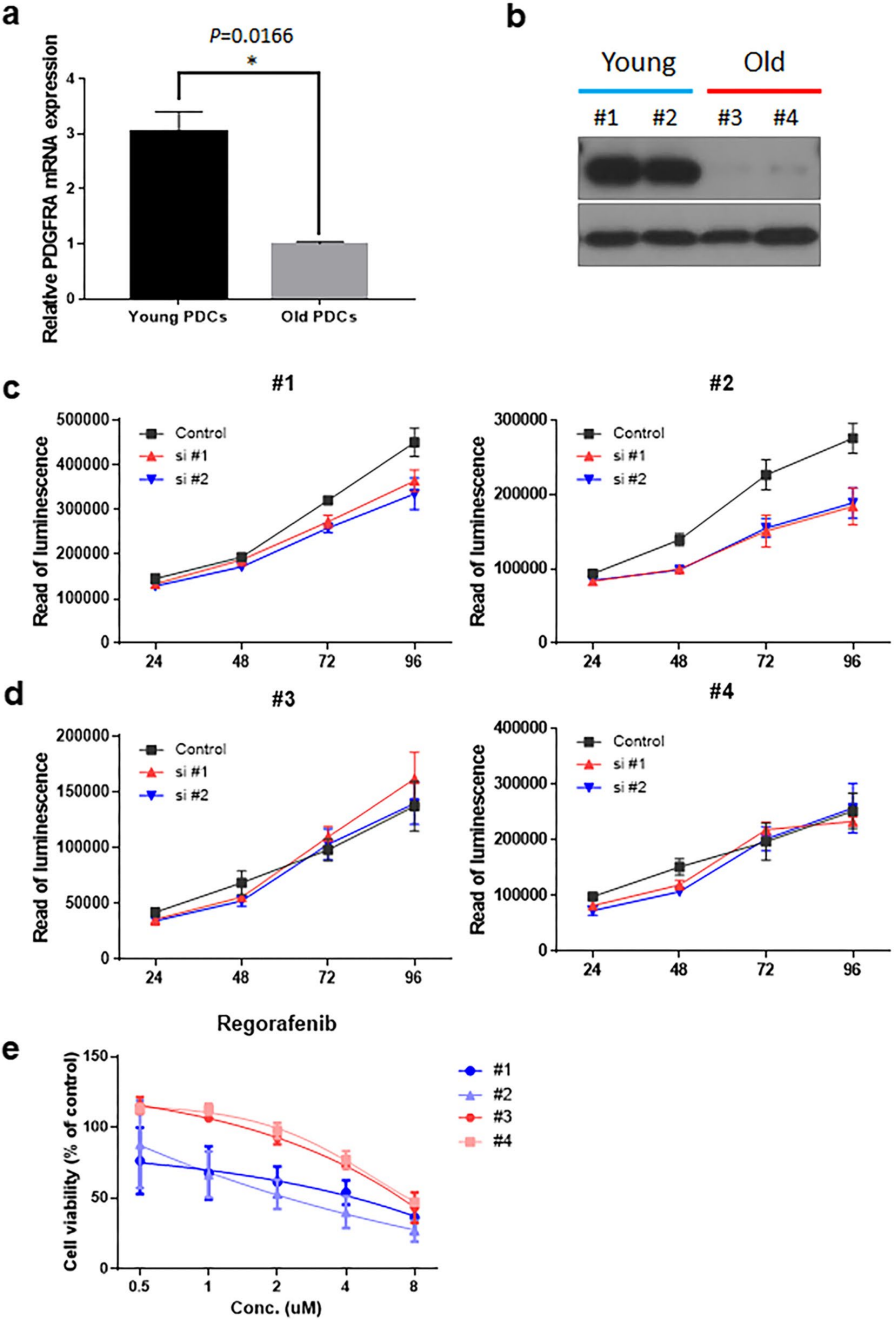
PDGFRA expression and PDGFRA-targeted drug response in PDC. **a** Boxplot between *PDGFRA* expression levels (RT-qPCR) in young and old PDCs. **b** Western blot of PDGFRA in young and old PDCs. **c** Viability by knockdown of PDGFRA in PDCs from young patients. **d** Viability by knockdown of PDGFRA in PDCs from old patients. **e** Sensitivity to regorafenib, an anti-cancer drug targeting PDGFRA, for PDCs. All patients used for PDC did not have any treatment before surgery

To investigate the key roles of PDGFRA in young CRC, we used PDCs derived from young and old age CRC patients that were not treated before surgery. (Table 4). The RT-qPCR analysis showed that PDGFRA expression was higher in young PDCs than in old PDCs (Fig. 5a, Additional file 4: Fig. S4a). Western blot analysis revealed that the expression of PDGFRA was higher in young PDCs than in old PDCs (Fig. 5b). To evaluate the possible roles of PDGFRA in young CRC, we transfected siRNAs in PDCs with two different siRNAs against PDGFRA to compare their proliferation ability using a Cell Titer GLO proliferation assay. Knockdown of PDGFRA in PDCs from young patients significantly reduced viability (Fig. 5c). However, PDCs from old patients did not alter viability compared to the control (Fig. 5d). To determine the PDGFRA-targeted drug for young CRC, we analyzed the correlation between drug sensitivity and gene expression [41]. The Cancer Cell Line Encyclopedia (CCLE) data indicate that sorafenib targeting PDGFRA only had a potential drug response in colorectal cancer (Additional file 4: Fig. S4b). However, our results showed that sorafenib did not show a distinguished drug response according to PDGFRA expression or age in PDCs (Additional file 4: Fig. S4c). Regorafenib is a small molecule multi-kinase inhibitor, including PDGFRA, and was recently approved by the FDA to treat patients with metastatic CRC [42–44]. To investigate the treatment strategy for young CRC, we used regorafenib in PDCs. PDCs from young patients were sensitive to regorafenib treatment, whereas PDCs from old patients were relatively resistant to regorafenib (Fig. 5e). When conducting HTS, the young age group was set to younger than 50 years old due to the small number of samples, and the results showed that the IC50 of regorafenib was significantly lower in the young group than in the old group (Additional file 4: Fig. S4d).

**Table 4:** Patient information in PDC

PDC	Age	Sex	Cancer type	Stage	Cell type
#1	40	M	Adenocarcinoma	IIIb	Poorly differentiated
#2	28	F	Adenocarcinoma	IIIb	Moderately differentiated
#3	83	F	Adenocarcinoma	IIIb	Moderately differentiated
#4	80	M	Adenocarcinoma	IIIb	Moderately differentiated

## Discussion

We first classified the CMS for young CRC patients and suggested a biomarker according to CMS. Moreover, we utilized PDCs to explore the function of the biomarker in young CRC patients. Previous studies on young CRC patients have focused only on a few cancer gene mutations and cancer-related gene expression or case reports, which were analyzed by categorizing patients into only two groups of young and old [45–49]. We divided the patients into young, middle-aged, and old age groups and focused on the CMS that gradually changed with age, as observed in a large amount of sequencing data of CRC patients. We found that the proportion of CMS4 gradually increased from the old group to the young group in the CRC patients. It is worth noting that our results demonstrated interesting findings that have not been previously reported in other studies on the CMS status of young CRC patients. From transcriptomic data, we found that PDGFRA*,* which is associated with CMS4, was significantly upregulated in young CRC patients than in old CRC patients. The protein level of PDGFRA was also higher in young CRC patients using PDCs. Additionally, when PDGFRA was downregulated by siRNA, the proliferation rate was significantly lower in young PDCs than in old PDCs. These results indicated that PDGFRA could possibly be a biomarker for young CRC patients.

We found that young CRC specific pathways include PDGFRA signaling, positive regulation of cell proliferation by VEGF-activated PDGF receptor signaling, cell chemotaxis, regulation of chemotaxis, positive regulation of PLC activity, and phosphatidylinositol phosphorylation. They are closely related to metastasis, angiogenesis, proliferation, and chemoresistance, which reflect the characteristics of the CMS4 type [19]. PDGFRA plays a major role in these pathways and is expressed in mesenchymal cells and epithelial cancer cells undergoing EMT [50]. PDGF, a ligand of PDGFRA, promotes cancer angiogenesis in the cancer stroma [51] and has positive regulation of cell proliferation and angiogenesis by VEGF-activated PDGFRA [52]. The chemotaxis of cancer cells plays an important role in cancer progression, metastasis, and drug resistance [53, 54]. PDGFRA promotes chemotaxis of cancer cells [53, 55, 56]. Activation of the PLC pathway increases cisplatin resistance and promotes cancer growth and metastasis [57–59]. PDGFRA induces activation of the PLC pathway and interacts with it [60–63]. These results suggest that PDGFRA may play a pivotal role in CMS4-associated pathways.

DNA sequencing data showed that TP53 mutations were frequently detected in the young CRC group than the old CRC group. A recent study reported that the TP53 mutation was also more frequent in young-onset CRC [64]. This was the same condition as the young CRC group that is < 40 years old [64]. Although both studies were consistent, the reason for a large number of TP53 mutations in young CRC patients remains unknown. In addition, the copy number at chromosome 5q22.2 was deleted only in the young CRC group. A high deletion rate in chromosome 5q22.2 may explain poor clinical outcomes in young CRC patients, which is because the region contains several tumor suppressor genes, such as *APC* and *MCC *[65–67]. Based on these results, more functional studies are required further study in TP53 mutation and deletion of chromosome 5q22.2 for young CRC patients.

Our data showed that regorafenib was more sensitive to PDCs of young CRC in which PDGFRA was upregulated. Regorafenib is a multi-kinase inhibitor [68, 69] that has been mainly indicated worldwide for patients with metastatic CRC [43, 69]. However, our data showed that regorafenib might be a potential candidate for treating young CRC patients. Therefore, regorafenib could be used for young CRC patients and for metastatic CRC.

## Conclusions

In conclusion, the CMS classification for young CRC patients may better inform clinicians of the prognosis and potential of novel therapeutic strategies. Our study suggests that CRC in young patients is associated with CMS4, PDGFRA, and CMS4-related genes. Our data will provide a greater understanding of young CRC patients and the prognostic value of biomarkers.

## Supplementary Information

**Additional file 1: Fig. S1.** Significantly changed pathways from DEGs. **a** Biological pathways from upregulated genes in young CRC patients. **b** Biological pathways from downregulated genes in young CRC patients.

**Additional file 2: Fig. S2.** PDGFRA expression between young and old CRC patients in TCGA dataset. **a** Scatter plot of *PDGFRA* mRNA expression and age. **b** Boxplot between PDGFRA expression levels in the young CRC group and in the old CRC group.

**Additional file 3: Fig. S3.** Co-expression of EMT and angiogenesis factor in TCGA. **a** Scatter plot of gene expression levels between PDGFRA and EMT-associated signatures. **b** Scatter plot of gene expression levels between *PDGFRA *and angiogenesis-associated signatures. **c** Scatter plot of mRNA expression of EMT markers and age. **d** Scatter plot of mRNA expression of angiogenesis markers and age. **e** Heatmap of co-expression patterns between EMT and angiogenesis markers.

**Additional file 4: Fig. S4.** PDGFRA expression and PDGFRA-targeted drug response in PDCs. **a** Boxplot of *PDGFRA* expression levels (RT-qPCR) for PDC samples (derived from young CRC: #1 and #2; old CRC: #3 and #4). **b** Prediction of drug sensitivity from gene expression data based on CCLE cell lines. **c** Viability by knockdown of PDGFRA in PDCs from young (#1 and #2) and old (#3 and #4) patients. **d** Comparison of sensitivity to regorafenib, an anti-cancer drug targeting PDGFRA between old and young PDCs.

## Data Availability

The data supporting the finding of this study are available within the article are available from the corresponding author upon request.

## Abbreviations

CRC: Colorectal cancer
CMS: Consensus molecular subtypes
MSI: Microsatellite instability
COAD: Colorectal adenocarcinoma
DEGs: Differentially expressed genes
TPM: Transcripts per million
FC: Fold change
EMT: Epithelial–mesenchymal transition
CCLE: The Cancer Cell Line Encyclopedia
