# Supplementary figures and images for: The role of PDGFRA as a therapeutic target in young colorectal cancer patients
Source: J Transl Med. 2021 Oct 26;19:446. doi: 10.1186/s12967-021-03088-7 (PMC8546951; doi:10.1186/s12967-021-03088-7)

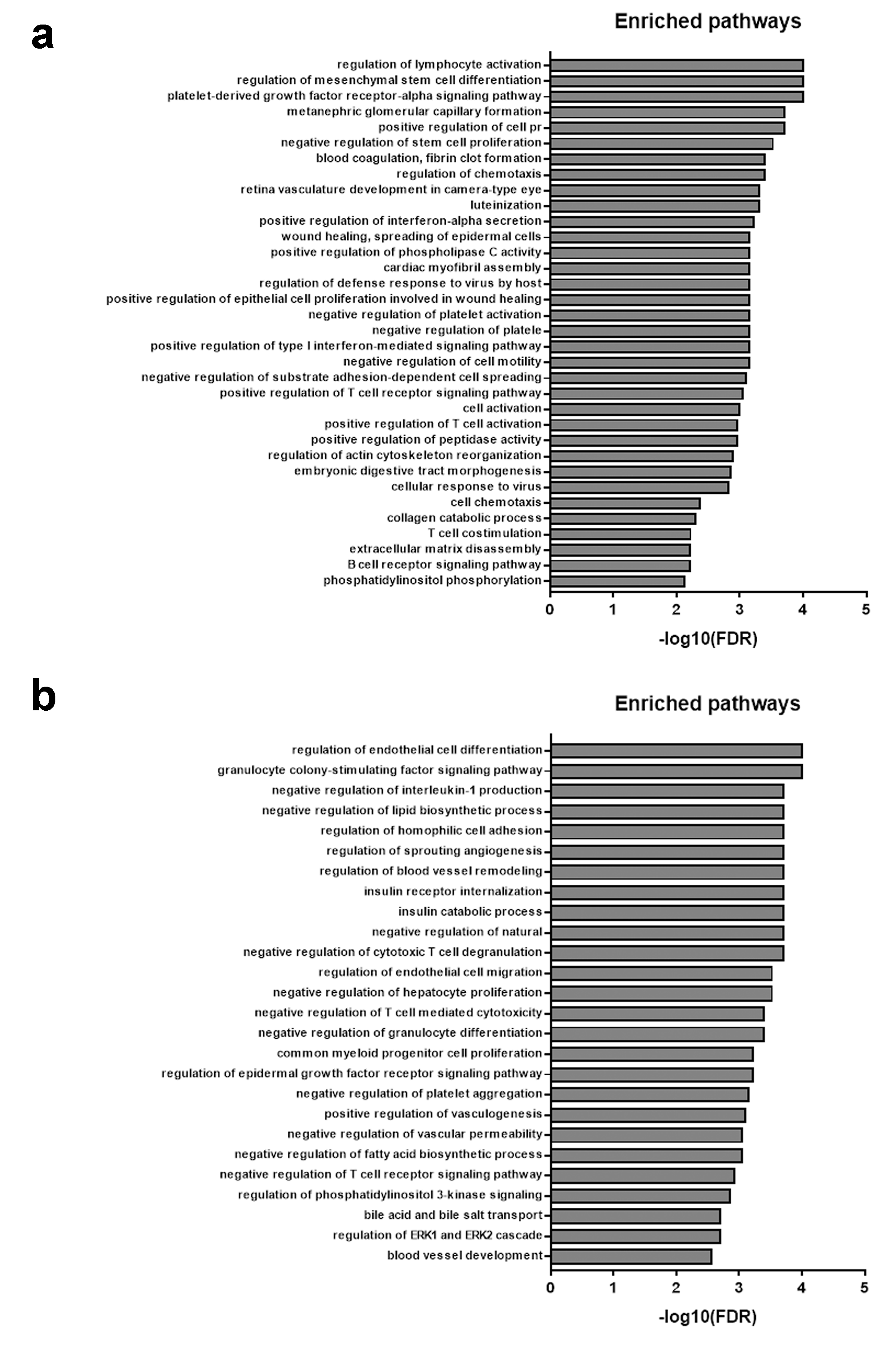

Supplement: Supplementary file 1 — Additional file 1: Fig. S1. Significantly changed pathways from DEGs. a Biological pathways from upregulated genes in young CRC patients. b Biological pathways from downregulated genes in young CRC patients. [file 12967_2021_3088_MOESM1_ESM.tif]

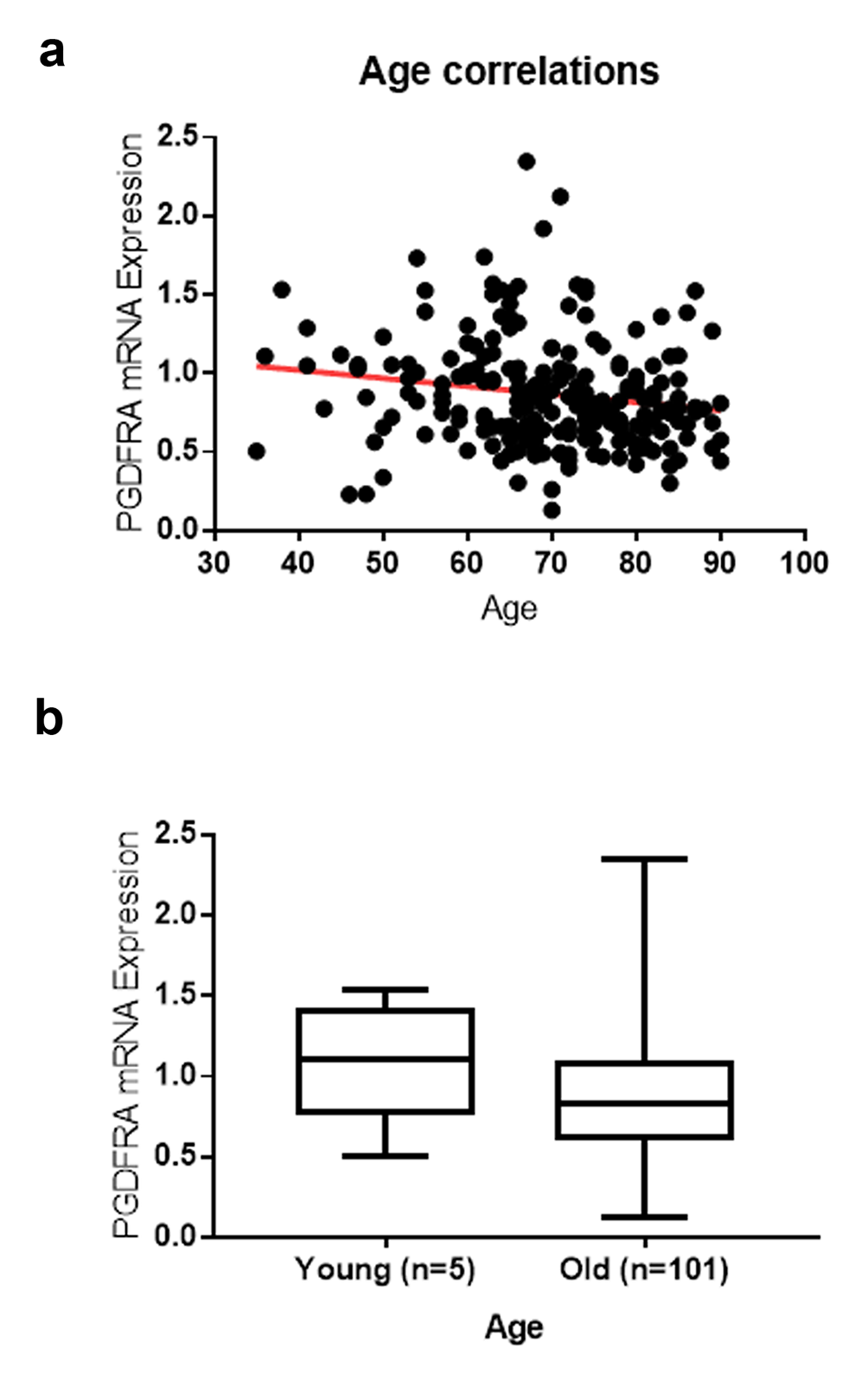

Supplement: Supplementary file 2 — Additional file 2: Fig. S2. PDGFRA expression between young and old CRC patients in TCGA dataset. a Scatter plot of PDGFRA mRNA expression and age. b Boxplot between PDGFRA expression levels in the young CRC group and in the old CRC group. [file 12967_2021_3088_MOESM2_ESM.tif]

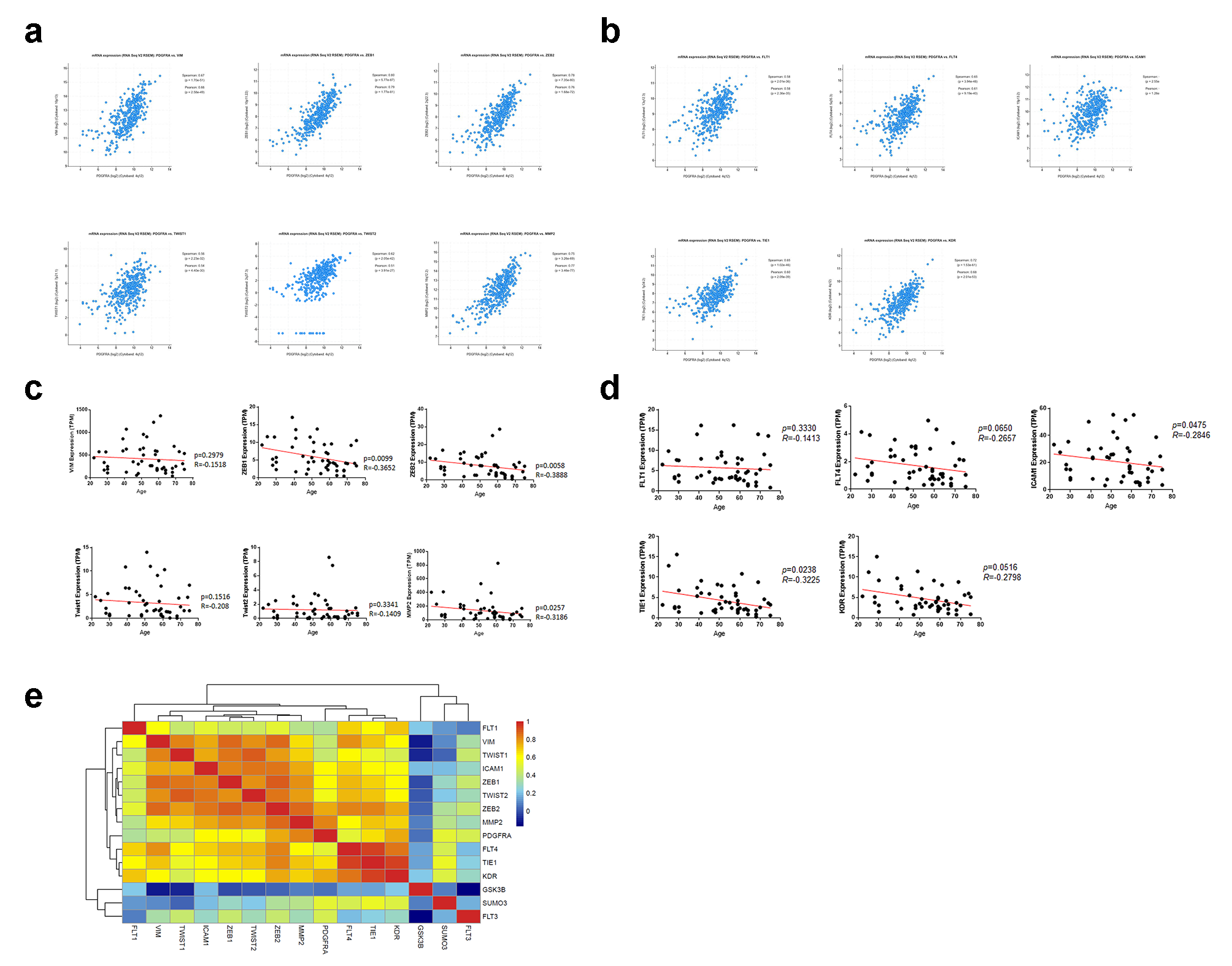

Supplement: Supplementary file 3 — Additional file 3: Fig. S3. Co-expression of EMT and angiogenesis factor in TCGA. a Scatter plot of gene expression levels between PDGFRA and EMT-associated signatures. b Scatter plot of gene expression levels between PDGFRA and angiogenesis-associated signatures. c Scatter plot of mRNA expression of EMT markers and age. d Scatter plot of mRNA expression of angiogenesis markers and age. e Heatmap of co-expression patterns between EMT and angiogenesis markers. [file 12967_2021_3088_MOESM3_ESM.tif]

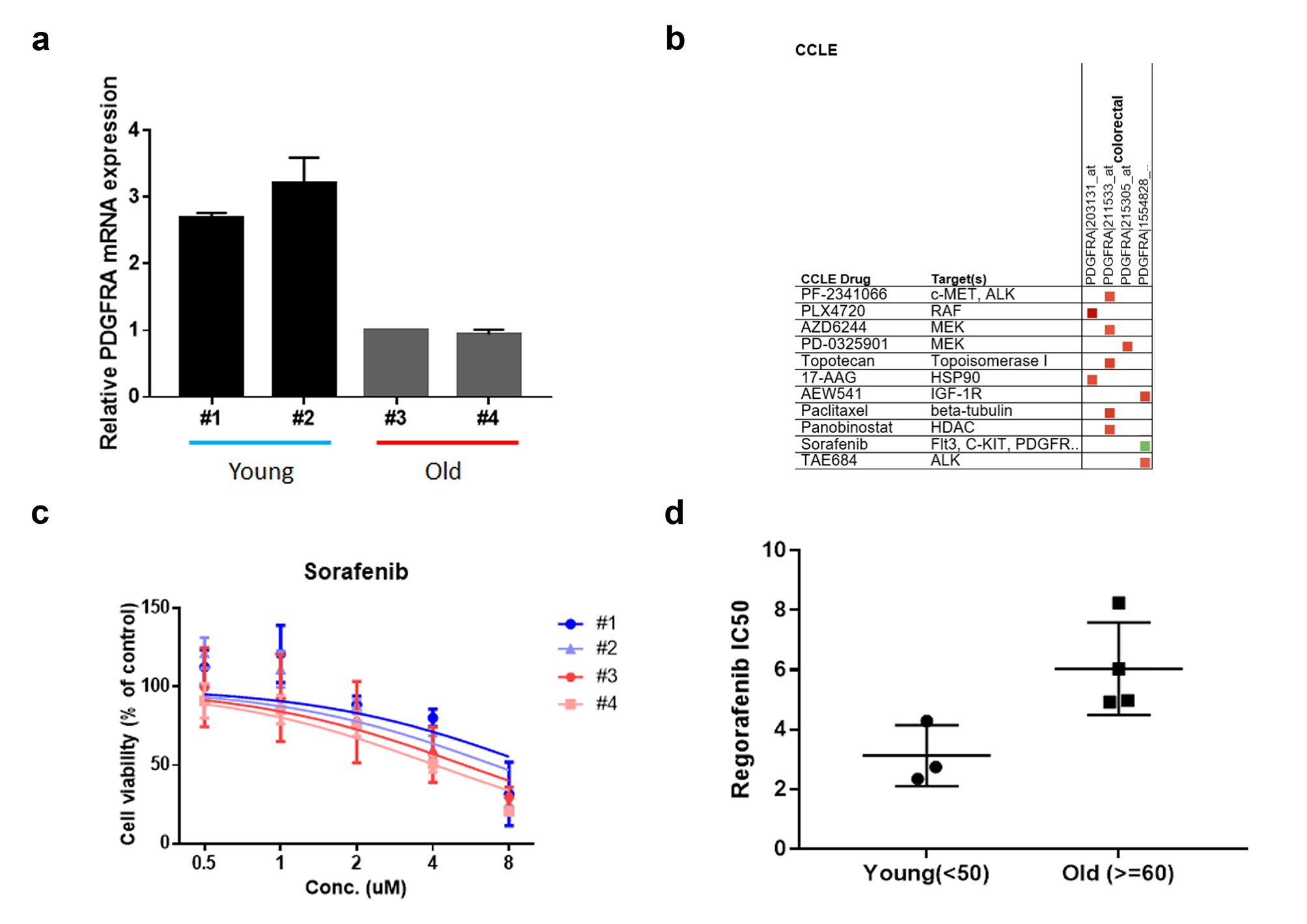

Supplement: Supplementary file 4 — Additional file 4: Fig. S4. PDGFRA expression and PDGFRA-targeted drug response in PDCs. a Boxplot of PDGFRA expression levels (RT-qPCR) for PDC samples (derived from young CRC: #1 and #2; old CRC: #3 and #4). b Prediction of drug sensitivity from gene expression data based on CCLE cell lines. c Viability by knockdown of PDGFRA in PDCs from young (#1 and #2) and old (#3 and #4) patients. d Comparison of sensitivity to regorafenib, an anti-cancer drug targeting PDGFRA between old and young PDCs. [file 12967_2021_3088_MOESM4_ESM.tif]
